# Cellular and humoral immune response between snail hosts and their parasites

**DOI:** 10.3389/fimmu.2022.981314

**Published:** 2022-11-10

**Authors:** Hanan Al-Khalaifah

**Affiliations:** Environment and Life Sciences Research Centre, Kuwait Institute for Scientific Research, Kuwait, Kuwait

**Keywords:** host, immune response, parasite, snail, mollusc

## Abstract

In invertebrates, the innate immune system protects against a wide range of microbiological infections. Several immunological processes are involved in the interactive immune response between snails and their parasites, including phagocytosis, nitric oxide synthesis, phenol oxidase activity, lysozymes, and lectin formation. The immunological responses connected to the interaction between snails and parasites are discussed in detail in the current research. Understanding the nature of these interactive reactions will enable scientists to explore approaches to eliminate and cure parasitic infections.

## Introduction

Parasitism is a symbiotic relationship between two species in which one species (parasite) is physically and physiologically dependent on the other (host) for a part of its life cycle. On the other hand, mutualism is a relationship in which both organisms gain some degree of benefit. Mutualism is usually temporary or not obligatory. Often the parasite initiates the onset of a disease. Pathogenesis is defined as the development of a particular disease, including the involvement of specific events, affected systems, damage mechanisms, and the timing of the course of the disease (1–6).

Many families of molluscs, such as the Planorbidae, and Lymnaeidae, serve as intermediate hosts for trematodes. Host-parasite relationships are too complex to generalize infectivity and pathogenicity (7). The Phylum Mollusca is the largest after that of Arthropoda. The most crucial taxonomic class is the Gastropoda, which has 40,000–150,000 living species today. The latter are infected with viruses, bacteria, and parasitic flatworms. These trematodes have a complex life cycle, including a gastropod as a transitional host. Sexual reproduction of trematodes in snails produces transmission stages that infect the host during the subsequent life cycle (8).

Asymmetrical molluscs with well-developed feet and radula are known as gastropods or snails. Their visceral mass is coiling in spirals. They can be found in freshwater, marine, and terrestrial settings. Prosobranchia, Opisthobranchia, and Pulmonata are the three subclasses that comprise the Gastropoda class. The Lymnaeidae family of snails belongs to the order Basommatophora and class Pulmonata, and includes the snail *Lymnaea stagnalis*. Freshwater scavengers like this snail search the bottoms of ponds, lakes, and marshes for food. It is a rather large species with thin and highly pointed spires. It can be found in Algeria, Morocco, North America, Europe, and Asia (9, 10). *L. stagnalis* generally favors slow-moving or stagnant waters and inhabits shallow ponds with dense vegetation feeding on algae or decaying plants. It is also predatory and sometimes consumes newts, small fish, or other snails. Because it is a pulmonate, it breathes through its lung by inhalation at the water surface. This feature allows it to thrive in oxygen-deficient environments (11).

The morphological and chemical protective barriers that compose the innate immunity of molluscs guard against infection by pathogenic bacteria and parasites, injury to the underlying tissues, and loss of bodily fluids. The main physical barrier is the mucus and shell with which mollusc soft bodies are covered. Blood clotting and wound healing promote the integrity of bodily coverings. Molluscs have internal defensive mechanisms that include phagocytosis, nodule development, encapsulation, pearl production, atrophy, necrosis, and tissue liquefaction, among other cellular reactions. The most frequent molluscan blood cell type involved in cellular defenses is called a granular hemocyte (8). Tiny invaders are destroyed *via* phagocytosis, in which lectins and by-products of prophenoloxidase system activation are involved. By nodule development or encapsulation, numerous giant invaders are destroyed, either cellular or humoral. Lysozyme activity, lectins, and the phenoloxidase system combine to create the humoral components of mollusc immunity (12).

Initial research revealed that although invertebrates, including the Mollusca (the second-largest animal group), have innate defenses, they lack the lymphocytic immune system characteristic of vertebrate immunology. In-depth investigations of a few bivalve and gastropod species continue to disclose new facets of molluscan immunity while acknowledging the reality of common and taxon-specific immunological traits and using cutting-edge cell and molecular research capabilities (13).

Immunoglobulins are not found in molluscs. Hemocytes are known to have essential functions in mollusc internal defense. *In vivo* and *in vitro*, hemocytes may easily phagocytose various biotic and abiotic particles. Additionally, humoral components may be crucial in the host’s defense (14).

Immunological studies in the freshwater snail *Biomphalaria glabrata*, which spreads the *Schistosoma mansoni*-caused human blood fluke, has received much attention from gastropods. There is limited research on other gastropods, such as the Lymnaeidae family, which transmits medically and veterinary-relevant parasites. *L. stagnalis* has been used as a model for studying numerous biological aspects, including ecological immunology (8).

The objective of the current research is to shed light on some factors related to the immunological response brought on by the interaction of the snail hosts and their parasites. The snails produce phenol oxidase, lysozyme, lectin, and phagocytosis as part of their innate immune response.

## The immune response of molluscs

Molluscan immunology has been investigated in a minority species of the phylum Mollusca. Many studies have focused on a few species of Gastropoda and recently on some species of Bivalves and Cephalopods due to their simplicity of collection, animal size, responsible animal husbandry, selective genetic lineage rearing and disease transmission, and economic importance. Preliminary experimental studies exposed the molluscs to inorganic material such as Indian ink and pathogens introduced by bacterial injection or by infection with human parasites such as *Schistosoma mansoni*. The snails were observed to be capable of clearing out bacteria from circulation (15). They could survive due to their high immunity and rapid parasite clearance after their first encounter (13). Other invertebrates, including molluscs, are well known to have an innate immune system to defend them against diseases and other invaders. A lot of evidence has proven the existence of immune components that can combat infectious elements (16–20).

Host-parasite interactions are usually very complex. The host recognizes the parasite as non-self and initiates host immunological reactions and immune signaling. Immune receptors detect the presence of parasites or foreign bodies. Innate immunity uses a few sensors to identify the chemical patterns linked to pathogens (PAMP). Molluscs do not have adaptive immunity and depend on innate or non-specific immunity for the host defense. They possess a wide range of high specificity innate immune receptors during immune recognition without the autoimmune cost in the absence of adaptive immunity (21).

### Anti-inflammation and immune modulation of some snails

The indirubin dye has the potential to track the hike in reactive oxygen species (ROS) from macrophage cells. This activity in relation to anti-inflammation has been reported for its brominated spinoffs in rat microglial and RAW264.7 cell cultures. Particularly, the RAW264.7 cell culture, the release of inflammatory cytokines, like interleukin (IL)-6 and IL-1β, was inhibited by indirubin. Similarly, anti-inflammation is also linked with an oxidation product oxidation product in the course of Tyrian purple production, isatin which inhibits formation of prostaglandin E2 (PGE2), cyclooxgenase 2 (COX-2), nitric oxide (NO), tumor necrosis factor-alpha (TNF-α), and inducible nitric oxide synthase (iNOS) in a lipopolysaccharide (LPS) and interferon gamma (IFN-γ)-stimulated RAW264.7 (22). The bioactivity of *Haliotis discus hannai* sp. includes inhibiting nitric oxide (NO) production in RAW264.7, decreasing tumor necrosis factor-*α* (TNF-*α*) and interleukin 6 (IL6) concentration, suppressing NO production through inducible nitric oxide synthase (iNOS), and decreasing the production of TNF-*α*, IL-6, and interleukin-1 (IL1*β*)) (23, 24). In *Haliotis diversicolor* there was a decrease in iNOS expression and increase in macrophage activity (25). There was reduction in the occurrence of hemolysis in *Neverita didyma* sp (26).. *Volegalea cochlidium* reduced occurrence of hemolysis, activation of phagocytosis at low concentrations, and repression of phagocytosis at high concentrations (26, 27). Reactive oxygen species (ROS), TNF- *α*, and NO production are inhibited by the species of *Filopaludina bengalensis*, as well as NF-B translocation (28). For the bioactivity of reduction in NO concentration and LOX inhibition, *Aplysia fasciata* and *Aplysia punctuate* sp. are utilized (29), *Dicathais orbita* NO production is inhibited, as well as the production of TNF- *α*, Nuclear factor kappa B (NF-B) translocation, and prostaglandin E2 (PGE2) (30).

### Phagocytosis

The immune system of invertebrates does not involve antibodies. Their defense line consists mainly of cellular defense; however, humoral factors are essential in getting rid of the infectious particles (31). The cellular factors include phagocytic cells capable of recognizing self-material from the non-self material. Phagocytosis and encapsulation are the primary mechanisms by which foreign particles are eliminated in molluscs. These mechanisms involve the role of agglutinins, opsonins, and lysozymes (32). Phagocytosis was first discovered by Stauber (33), who demonstrated that India ink was phagocytosed by cells of *Crassostrea virginica*, followed by its rejection from the mollusc body *via* the epithelial tissues. The first line of defense against invasive or established organisms and particles is thought to be the phagocytic cells in the hemolymph of gastropods. These hemocytes circulate within the hemolymph and reside in the connective tissues (34). Cells lining the hemolymph spaces can trap microorganisms. Cells like phagocytic reticulum cells in tissues are also a part of the defense system (35).

Ultrastructural studies have revealed that hemocyte phagocytosis occurs in two ways. The first one is the formation of pseudopodia that can extend to engulf foreign particles or invaginations of the plasma membrane. In both cases, phagosomes are formed. These phagosomes are fused with lysosomes in the cytoplasm that contain enzymes such as peroxidase and acid phosphatases to hydrolyze the engulfed particle or organism. Interestingly, fixed hemocytes in connective tissue can also phagocytose foreign particles (36). The ability of molluscs to discriminate between viable and non-viable parasites reflects the complexity of the host-parasite relationship.

Many types and numbers of hemocytes in molluscs have been determined through investigations. However, given these hemocytes’ diversity in morphology and function, it remains a contentious issue. These cells take the role of digestion, excretion, wound healing, shell repair, transport, and encapsulation, in addition to phagocytosis (37).. In molluscs, morphological characteristics such as cell size, the ratio of the nucleus to the cytoplasm, the shape of the nucleus, and the presence of granules in the cytoplasm are used to categorize hemocytes. Depending upon the species, molluscs can have a single or several categories of hemocytes. These cells may have been derived from connective tissue or specialized organs known as an amoebocyte-producing organs (APO) in gastropods (13). Studies have revealed the presence of two types of hemocytes: granulocytes and hyalinocytes (38).

Additionally, granular cells were detected together with acidophilic and basophilic granulocytes. The microscopic cells are less than 8 m in size, whereas the agranulocytes, also known as hyalinocytes, are more giant spreading cells with pseudopodia and a polymorphic nucleus. The hemolymph of molluscs has large spreading cells in the majority (39).

However, recently hemocyte subtypes are further divided into more varying populations based on numerous criteria researchers have set for distinct bivalve species (40). As a result, it is challenging to compare or extrapolate the results of molluscan investigations. The presence of the hemopoietic organ in molluscs is not regular. Hemocytes can be formed in other ways, such as spontaneous mitosis of hemocytes that can increase when blood flows through sinuses, soft tissues, and hemolymph vessels. This could induce plasticity during hemocyte maturation instead of cell categorization into distinct subtypes. Typically, the presence or absence of cytoplasmic granules is the determining factor for hemocyte classification of hyalinocytes and granulocytes. These two types of cells have been reportedly found in *Crassostrea gigas, Biomphalaria glabrata, Ruditapes decussatus, Mytilus edulis, Tapes philippinarum*. Morphologically, hyalinocytes may also be separated into giant hyalinocytes with small nuclei and small hyalinocytes with large nuclei. Characteristic features of granulocytes include their effective phagocytosis of microbes, production of hydolytic enzymes, expression of reactive oxygen species (ROS), and support for internal cell death. However, The molecular process underlying the functional differentiation of hemocytes is still unexplainable (41).

It’s interesting to note that the transitional period between round and spreading cells is middle morphological. Some studies have asserted that younger cells are compact and rounded, whereas older cells have enormous spreading and have high phagocytosis capacity (42). For example, the small round cells in *L. stagnalis* carry less phagocytosis than the spreading cells.

Tripp and Kent (43) have shown that hemocytes can eliminate 90 percent of invading germs *in vitro* after 24 hours and 99 percent after 72 hours. It was proposed that the cells’ glycolytic process would provide energy to hemocytes for phagocytosis. It was proved by Cheng (44), who showed that the hard clam *Mercenaria mercenaria’s* hemocytes consume glucose and glycogen and generate lactate devoid of increasing oxygen consumption.

A phagocytic cell is a typical hemocyte in *L. stagnalis*. Scientists observed different morphologies of their differentiated stages, which are mainly affected by the age of the cells. Young cells (8 µm in diameter) are less differentiated and have a high nucleus to cytoplasm ratio, while large cells (20 µm) are more differentiated with a lower nucleus to cytoplasm ratio. Different subpopulations of hemocytes can be isolated and distinguished using various techniques like agglutinins, monoclonal antibodies, and isopycnic centrifugation (35). Scientists like Krupa and Lewis (45) and Harris (46) reported the presence of granulocytes and hyalinocytes in *L. stagnalis*. Others, like Sminia (36), reported only one kind of hemocyte, the amoebocyte. Phagocytosis is divided into four steps: 1-attraction between the phagocyte and the non-self-particle, 2-attachment of the non-self-particle to the phagocyte surface, 3-internalization, and 4-internal degradation (47).

The number of hemocytes in molluscs varies considerably between species and intraspecies. Sminia (36) reported that the average number of cells in the hemolymph of *L. stagnalis* is 0.5–4 × 10^6^ per ml. The total number of cells is affected by many factors such as the collection method, site of puncturing, size of mollusc, temperature, infection, and type of wound (48). Feng et al. (49) have also reported that the circulating hemocytes are withdrawn during snail bleeding, and their number is affected by temperature. In *Bullia laevissima*, the cell count is higher for hemolymph obtained from the heart and arteries than for veins (50).

There are considerable differences in the number of circulating hemocytes in the hemolymph due to environmental conditions and different species of molluscs. Their numbers are also affected by the location of the hemolymph sample; for example, the sample taken from *B. glabrata* during foot discomfort has a more significant number of hemocytes than that acquired in normal situations. Other factors include the animal’s age, the presence of parasites, and the amount of water in the hemolymph, the organism’s general health, and the time of year (active or hibernation). Higher circulating hemocyte levels are present in parasite-infected patients compared to non-infected molluscs. Hemocytes can also aid in wound healing, shell formation, transport of calcium-rich deposits, mineral ions, and calcium-binding proteins (51).

Sminia (36) also reported that large specimens (30 mm) have about 2–3 times higher cell numbers than the smaller ones (10 mm). An increase in cell number after one hour of bleeding was also observed in *L. stagnalis*. This increase suggested a rapid release of hemocytes from the connective tissue into the hemolymph circulation upon bleeding (52).

### Encapsulation

A fibrous capsule in encapsulation can surround foreign particles or parasites. It is caused by infiltration and aggregation of hemocytes at the site of infection when the infectious element is too significant for phagocytosis. This initiates the appearance of fibroblast-like cells that produce collagen and contribute to capsule formation (37). Studies have revealed that encapsulation in molluscs is implausible in the case of viable parasites. Pan (53) reported encapsulation occurs in snails infected with schistosomes when the cercariae degenerate, while viable cercariae and sporocysts remain unencapsulated.

On the other hand, thick capsules are formed around viable parasites in non-susceptible strains of molluscs (54). If the non-self-particles are situated on the nacre-secreting mantle, a pearl is formed in a process called maceration (47). Encapsulation is a defense mechanism triggered by detecting non-self and protease cascades by toll-like receptors (TLRs). During protease cascades, the enzyme phenol oxidase causes the oxidation of polyphenols with or without dopamine as a substrate to encapsulate non-self in a rigid structure made of melanin. To avoid implications from the phenol oxidase cascade, these reactions are under strict regulation of the prophenoloxidase (proPO) activation system (55).

### Lectin production

Lectins are found in plants, bacteria, fungi, and animals. They are non-enzymatic and non-immunoglobulin carbohydrate-binding proteins that reversibly attach to particular carbohydrate structures, either free in solution or on cell surfaces. They are frequently categorized for their specificity of saccharides. The carbohydrate specificity of a lectin is defined as the fact that monosaccharides or oligosaccharides inhibit the lectin-induced precipitation or agglutination responses. Similar lectins, like galactose-specific lectins, exhibit significantly distinct sugar-binding preferences. More and more lectins that never show a high affinity for simple saccharides are being discovered. Lectins are considered to be multivalent, having two or more sugar-binding sites. They play an important role in cell recognition and have various physiological functions. These enabled scientists to study cell histochemistry, differentiation, biochemical pathways, typing cells, and stains (taxonomy) (56).

Lectins from bivalve molluscs are known to be polyreactive. They are composed of many subunits of different masses and affinities that widen the spectrum of monosaccharides capable of binding to them. Moreover, lectins are also composed of identical subunits which bind to different monosaccharides (57). Hemolymph from *Modiolus* contains three types of subunits, while *Anadara granosa* contains two subunit types (56).

On the other hand, lectins from gastropods are oligoreactive, meaning they have a very narrow specificity to oligosaccharides. In both systems, lectins eliminate foreign particles and organisms from molluscs (58). Thus, they play a significant part in non-specific immune response.

Pattern recognition receptors (PRR) for lectins and carbohydrates recognize distinct PAMPs and other microorganisms, including bacteria that have carbohydrates and glycoproteins on their cell walls. They bind, agglutinate, and opsonize microorganisms to promote phagocytosis and eradication. Molluscs are the primary hosts of the c-type (calcium-dependent) lectin. This type of lectin agglutinates bacterial cells and is upregulated due to bacterial challenges in some molluscs, such as *Cassostrea farreri* and *C. virginica*. Galectins from *C. virginica* are a family of lectins that can bind to β-galactosides and recognize *P. marinus* that uses these galectins to enter the host cell. After infection with *P. marinus*, galectin’s down regulation might indicate the host’s attempt to restrict infections (21).

Conjugated lectins are used in cell or tissue surface membranes. Enzymes, biotin, and FITC are used as markers for lectins. These can also be detected by radioactive means using Iodine. Moreover, electron-dense means are used when the labeling agent is colloidal Au ferritin.

Fluorimetry, light, ultraviolet (UV), and electron microscopy techniques are used to detect the fluorescence of the lectin labels. FITC and TRITC are frequently used as fluorescent labels. Many lectins labeled with markers are commercially available (59).

The lectin-binding phenomenon has been extensively utilized in histochemistry for research and therapeutic applications to identify particular carbohydrates and derivative structures such as cells and tissues, including glycolipids and glycoproteins. These also purify and isolate carbohydrates from particular cells, including bone marrow and lymphocytes. The analysis of cell membrane architecture, glycosylation processes, differentiation, cell division, growth, developmental changes, and the mapping of neural connections are other significant tasks that can be accomplished with the aid of lectins (60).

It is well known that the bodily fluids of invertebrates include a variety of lectins, preferably as body-protective elements (59). Mollusc hemolymph has been reported to react and agglutinate many cells. This indicates the presence of hemagglutinins (i.e., lectins), which are defense mechanisms against foreign particles and parasites by acting as opsonins to increase their ingestion and phagocytosis by hemocytes. Furthermore, their activity plays a significant role in nodule formation in other invertebrates (61). It was reported that hemolymph from the freshwater snail *Viviparus molleatus* agglutinates red blood cells from rabbits (62). Opsonic factors in the hemolymph of *Helix aspersa* and *Biomphalaria glabrata* are well reported (63).

Furthermore, increased phagocytosis of bacteria was observed in oyster hemocytes when the bacteria were coated with lectins (64). Uhlenbruch et al. (65) have shown that the hemolymph of *H. pomatia* and *H. aspersa* could agglutinate human cells. Hemagglutination of vertebrate erythrocytes by oyster hemolymph, *Crassostrea virginica*, has also been reported (66).

Numerous genome and transcriptome studies determined that within the same lectin family, the sequence variety, sugar-binding abilities, and carbohydrate recognition domain (CRD) of molluscan lectin-like molecules are significantly different. The high molecular diversity and plasticity of CRDs permit broad-spectrum recognition in invertebrate lectins. All prominent lectin families have different sizes and diversities between bivalve and gastropod molluscs. It might be due to environmental factors and adaptation to microbiomes and pathobiomes. For example, many C1q domain-containing (C1qDC) protein variants are present in bivalves compared to a few gene copies in gastropods (67).

Fluorescein isothiocyanate (FITC)-labeled lectins were used to examine the presence of different kinds of lectins on the hemocyte’s surface. These labeled lectins have extensive applications in direct labeling techniques. Conjugating lectins with fluorescent labels are accessible; however, the fluorescence fades with time. So, the measurement should be done very quickly. Storage at 4° C in the dark might help in this case. Moreover, identification of the cell type became difficult because the tissues shine against a black background. Thus, samples in such an experiment were examined directly after preparation accordingly (68).

Enzyme labels are also widely used. The most common problem with using enzyme-conjugated lectins is that some tissues contain endogenous enzymes that might react the same way as the label, giving the same color. For example, the kidney is extremely rich in alkaline phosphatases, and the spleen is rich in peroxidases. Therefore, different enzymes should be used in such cases to avoid misleading results (69).

Hemocytes of mollusc *L. stagnalis hemocytes* have sugar moieties on their surface that bind to various lectins. This binding can be inhibited or reversed by adding lectin-specific sugars.

Interestingly, Georgieva et al. (70) used the lectin binding assay to study surface carbohydrates’ existence and distribution in the tissues of uninfected or infected *Galba trunculata* snails with *Fasciola hepatica*. The authors attempted to find some similarities to represent mimicry of the evasion strategy used by the of snail-trematodes system. However, the authors found variations in the host and snail pathogenic larval stages of *F. hepatica*, binding patterns in the head-foot mantle, hepatopancreas, genital glands, and Reno pericardial complex. Infection with *F. hepatica* led to changes in the binding pattern of head-mantle cells and *Arachis hypogaea* in the tubular epithelium of hepatopancreas observed with Glycine max labeling (70).

In addition to agglutinins, hemocytes of molluscs are also known to produce toxic metabolites such as lysosomal enzymes, other lysins, nitric oxide (NO), and phenol oxidase (71). These metabolites help in the process of parasite elimination.

### Lysozymes

Lysozymes are enzymes that break down bacterial cell walls by hydrolysis action. They can be found in sweat, egg whites, saliva, plant tissues, and tears (72). They are a classic mollusc immune effector of innate immunity. Lysozyme is a bacteriolytic enzyme produced by various organisms, including bacteria, bacteriophages, fungi, plants, and animals. Lysozymes have been associated with tumors and perform other features, including digesting, antiviral activity, and anti-inflammatory (73). The concentration of lysozymes in the hemolymph of molluscs was reported to change according to an immune response. Feng and Canzonier (74) have shown a significant increase in lysozymes titers in the hemolymph of oysters infected with *Minchinia* sp., indicating these enzymes’ role in infection. It was discovered that *L. stagnalis* hemocytes have selective bacteriostatic activity, a defense against foreign substances/pathogens. Lysozymes aid in the detection of non-self-agents in addition to eliminating pathogenic components (75).

The beta-1, 4-glycosidic bond between N-acetyl-D-glucosamine and N-acetylmuramic acid in the peptidoglycan layer can be broken by the hydrolytic activity of the lysozyme, an essential antibacterial protein. It was reported that six lysozymes, which are g-type and i-type, are found in molluscs (76).

In addition, seasonal fluctuations in the lysozyme contents were reported (77). Oyster hemocytes produced more lysosomal enzymes in response to infection with the *Perkinsus marinus parasite* in winter than in summer.

Interestingly, Guo and He (76) identified and studied a g-type lysozyme from the sewage snail *Physa acuta*. These snails were collected from a river in China and transferred to the laboratory, where a full-length cDNA of a new g-type lysozyme (PALysG) was identified.

There are six types of lysozymes categorized according to structural differences. Lysozymes serve as innate immune protectors against exogenous microbial invasion, as well as having a role in digestive functions.Chicken-type (c-type), goose-type (g-type), plant, bacterial, T4 phage, and invertebrate-type (i-type) lysozymes are among those with catalytic, digestive and immunological properties. Three types of lysozymes have been detected in molluscs: c-, g-, and i-type. The c-type was detected in a chicken egg, the g-type was detected from the egg whites of the Embden goose, and the i-type was obtained from the starfish *Asterias rubens*. They play a significant role as antibacterial and immune-modulating agents. In addition, they are essential digestive enzymes in some animals. In molluscs, three types of lysozymes were detected in their digestive systems. The digestive gland is a crucial lymphoid site in molluscs, wherein the hepatopancreas can serve as a major site for the production of lysozymes (73).

### Phenol oxidase (tyrosinase)

Phenol oxidase (PO) is also regarded as a first line of defense in the molluscan initial immune response. It contributes to the production of melanin and tanning. Because little amounts of chemicals like lipopolysaccharide, peptidoglycan, and beta-1, 3-glucans from bacteria can enable its enzymatic response to occur, it is regarded as a non-self recognition system (78).

Tyrosinase, commonly known as polyphenol oxidase, is a copper-containing monooxygenase enzyme present in plant and animal tissues. It catalyzes the production of melanin and other pigments from tyrosine by hydroxylation of monophenols and the oxidation of o-diphenols to o-quinols, such as the blackening of a peeled banana exposed to air (79). PO binds to two copper ions (CuA and CuB), each ion bound by three conserved histidine residues (80).

PO is found in an inactive state called prophenoloxidase (proPO) in the hemolymph and is activated through some microorganisms’ lipopolysaccharides and 1,3-glucans (LPS). These molecules proPO can be converted to PO by endogenous serine proteases. Tyrosine is one of the monophenols catalyzed by PO to be converted into o-diphenol (DOPA or DOPAmine), which is then further oxidized to produce o-quinones (DOPA aquinone and DOPA aminequinone). The end product is melanin, contributing to pathogen asphyxiation. Melanization occurs with PO and requires activated proPO in invertebrates. The melanin deposits around or within the pathogen during oxidation and polymerization of phenols. Many cytotoxic molecules are produced during this process, including reactive oxygen (ROS), nitrogen (RNS) species, and quinoids, the melanin intermediates. Furthermore, an elevation of nitric oxide (NO) occurs in immunoreactive hosts, an effective effector molecule against invasive organisms. In one study, PO activity was significantly higher in dicrocoeliid trematode larvae than in the non-infected *Helix lucorum* (81, 82).

Hemocytes were found to enhance their peroxidase activity in *L. stagnalis* infected with the bird’s schistosome *Trichobilharzia ocellata* in the 2nd and 8th week after infection (83, 84). Adema et al. (85) have reported that this toxic metabolite can kill trematode larvae. Studies have shown that the production of this metabolite was reduced in hemocytes from *Biomphalaria glabrata* when treated with excretory/secretory products from *Schistosoma mansoni*. Connors et al. (86) have reported the presence of a molecule in these excretory/secretory products that cause this reduction. This PO reaction is also essential for wound healing and encapsulation of foreign particles (87).

PO enzymes are crucial to the process of reproduction, tissue pigmentation, and wound healing. Since they are the last component of the proPO activating system, they are also part of the inherent immune protection from invasive infections. These enzymes, which contain copper, are divided into three groups: I catecholases, which oxidize o-diphenols; (ii) laccases, which oxidize o-diphenols; and (iii) tyrosinase, which catalyze the hydroxylation of monophenols and oxidation of o-diphenols, p-diphenols, and p-diamines. All three PO actions are present in invertebrates. While tests without exogenous serine proteases quantify PO activity during infection, trypsin enzymes can measure all PO activity that is present in an individual. The assays are carried out in the presence of exogenous serine proteases (88).

Vorontsova et al. (89) analyzed the hemocytes and hemolymph of *L. stagnalis* to detect PO activity. No PO activity was observed in the hemocytes, while low activity levels were noted in the hemolymph without cells. When a specific PO inhibitor was added, it showed no effect on enzyme activity in the hemolymph, but the addition of hydrogen peroxide increased the activity. Le Clec’h et al. (88) designed a study to characterize PO activity in *Biomphalaria* spp. and the impact of *S. mansoni* infection. They used spectrophotometric assays using three specific substrates as PO inhibitors to determine PO activity in two kinds of *Biomphalaria* snails’ hemolymph. They also determined the impact of the parasite *S. mansoni* on the PO activity of its *B. glabrata* vector. It was concluded from the study that *S. mansoni* had a severe impact on PO production after six weeks of infection.

### Nitric oxide

The cardiovascular, reproductive, neurological, and immunological systems use the intercellular signaling molecule nitric oxide. In vertebrates, nitric oxide is produced by nitric oxide synthase, which converts arginine (Arg) to citrulline (Cit) in the presence of NADPH. The neural tissues of the central nervous system are also known to contain high nitric oxide levels. The oxidation reaction carried out by nitric oxide synthase involves the guanidine group of L-arginine, the consumption of five electrons, and the formation of nitric oxide as a final product (90).

NO is the nitrogen intermediate produced by NOS (nitric oxide synthase) isoforms that oxidize the guanidino nitrogen of L-arginine, leading to the production of equal amounts of L-citrulline and NO. NOS isoforms have also been identified in molluscs. mRNA encoding nNOS (Lym-nNOS) has been cloned and sequenced from the central nervous system of *L. stagnalis*, the key modulatory neurons of the feeding network (91). In mammalian cells, nNOS/eNOs and iNOs expression is regulated by signal transduction pathways. The generation of NO in response to an immunological challenge has been described in hemocytes from a few molluscs such as *Mytilus galloprovincialis*, *Ruditapes decussatus*, *Crassostrea gigas*, and *Viviparus ater*. One study investigated the role of cell signaling pathways of primary hemocytes from *L. stagnalis* in NO production. Protein kinase (PKC) and extracellular signal-regulated kinase (ERK) were concluded to be a part of the signaling machinery that regulates NOS activation and NO production in molluscan hemocytes (92).

Gourdon et al. (93) have reported the presence of NOS in hemocytes of *Mytilus galloprovincialis* and its involvement in the non-specific immune system. Nitric oxide synthase was also located in the nervous system of *L. stagnalis*. Intense staining at different nerve fiber locations indicated this compound’s presence (94).

NO has been widely studied in the phylum Mollusca and acts as a negative regulator of metamorphosis, as observed in the Pacific oyster *Crassostera gigas*, the eastern mud snail *Ilyanassa obsoleta*, the slipper shell snail *Crepidula fornicata*, and the sea slug *Alderia willowi*. However, NO has also been linked to other molluscan processes, including growth, neurotransmission, the immune system, feeding behavior, chemosensory activation, olfaction, and stress response (95, 96). Some parasites, including flatworms and trematodes, have produced NO. Schistosomes produced NO during their feeding and defense mechanisms (90). Therefore, it was speculated that some of the NO might come from the parasite in the case of live parasite inoculation.

Furthermore, given that NO synthesis rises in response to infection with living, dead, and parasite products, it may be deduced that it is essential for the defense against them. Nitric oxide synthase was found in the hemocytes of *Mytilus galloprovincialis*, and Gourdon et al. (2001) described its role in the non-specific immunological response.

Inspite of the snail’s immune response mentioned above, the process of parasite development in the snail depends on many factors such as the parasitic capacity to locate and enter the snail, adaptation to stressful conditions, obtaining nutrients for growth and reproduction, and overcome the host defenses (97). Snails fail to eliminate parasites because these parasites are very efficient in evading the host’s immune system by using snail-like or snail-derived molecules akin to molecular mimicry (98). In addition, the trematode parasite may suppress the immune defense of the snail, especially if there is a multi-parasitic infection. The parasite’s excretory/secretory products induce the production of immune regulatory proteins from the snail’s nervous system (99). Lie et al. (100) have reported that *B*. *glabrata hemocytes* infected with *E. paraensei* lose their ability to encapsulate trematodes due to parasite interference with the snail’s immune system. However, these hemocytes were able to phagocytose other foreign particles.

## Conclusions

Molluscs, including gastropods, have an effective immune defense against foreign particles, invading microorganisms, and parasites. This defense system is a part of an innate immune response consisting of cellular and humoral actions. It includes phagocytosis, agglutination, encapsulation, and toxic metabolites like nitric oxide and phenol oxidase.

## Author contributions

The author confirms being the sole contributor of this work and has approved it for publication.

## Acknowledgments

The author would like to extend her sincere thanks to the management of Kuwait Institute for Scientific Research (KISR) for its continuous technical and financial support.

## Conflict of interest

The authors declares that the research was conducted in the absence of any commercial or financial relationships that could be construed as a potential conflict of interest.

## Publisher’s note

All claims expressed in this article are solely those of the authors and do not necessarily represent those of their affiliated organizations, or those of the publisher, the editors and the reviewers. Any product that may be evaluated in this article, or claim that may be made by its manufacturer, is not guaranteed or endorsed by the publisher.
